# Expression of ultralong complementarity determining region 3 and development of IgM and IgG B cell receptor repertoires in Holstein heifer calves

**DOI:** 10.1093/immhor/vlaf079

**Published:** 2026-01-14

**Authors:** Tess E Altvater-Hughes, Harold P Hodgins, Douglas C Hodgins, Cathy A Bauman, Bonnie A Mallard

**Affiliations:** Department of Pathobiology, Ontario Veterinary College, University of Guelph, Guelph, Ontario, Canada; Department of Biology, University of Waterloo, Waterloo, Ontario, Canada; Department of Pathobiology, Ontario Veterinary College, University of Guelph, Guelph, Ontario, Canada; Department of Population Medicine, Ontario Veterinary College, University of Guelph, Guelph, Ontario, Canada; Department of Pathobiology, Ontario Veterinary College, University of Guelph, Guelph, Ontario, Canada

**Keywords:** BCR repertoire, BCR development, bovine, ultralong complementarity determining region 3

## Abstract

There are currently knowledge gaps regarding the development of the bovine B cell repertoire especially for cells expressing B cell receptors with heavy chain ultralong complementarity determining region 3 (CDR3). The objective of this study was to assess percentages of B cells with ultralong CDR3s, and immunoglobulin heavy chain gene usage in Holstein calves from birth to 10 mo of age. Blood was collected from 7 heifer calves on the following days of life: 0 (prior to ingestion of colostrum), 2 (after colostrum ingestion), 42, 56, 90, and 285. IgM^+^ and IgG^+^ B cells were collected using magnetic-bead separation. RNA was extracted, cDNA was produced, and IgM and IgG sequences were amplified using polymerase chain reactions. Amplicons were sequenced using long-read Oxford Nanopore sequencing on R10.4 flow cells. Differences in the percentages of B cells with ultralong CDR3s were assessed using non-parametric Wilcoxon signed-rank tests. The mean percentage of productive IgM ultralong CDR3 sequences at birth was 3.27%, which decreased by day 42 and remained low until there was a significant increase between day 90 and ∼day 285 (5.68% ± 1.51 standard error of the mean, *P* = 0.03). The mean percentage of productive IgG ultralong CDR3 sequences was low at birth (0.89%) and remained low until there was a significant increase between day 90 and ∼day 285 (6.86% ± 0.99, *P* = 0.03). These increases in the percentages of sequences with ultralong CDR3 may be indicative of increased antigenic exposure and concurrent maturation of immune functionality.

## Introduction

Bovine calves are born with functionally immature immune systems and typically lack antigenic exposure to pathogens prior to birth.^1^ There is a lack of transfer of maternal antibodies across the placenta to the fetus.^2^ Instead, maternal antibodies are transferred to the calf through the first milk, known as colostrum. Components transferred via colostrum to calves include immunoglobulins (Ig) (especially IgG1), cells, growth factors, and nutrients.^2^ These colostral components are either derived from the dam’s circulation or are produced locally in the mammary gland.^3^ Consumption of colostrum by the calf in the first hours of life leads to absorption of components across the gut epithelial lining, into the circulation.^2^

Neonatal dairy calves experience many different stressors in the first months of life. From the moment of birth, the calf is exposed to a multitude of new pathogens and microbes, with the birthing process being stressful in itself.^4^ Calves must then consume colostrum to absorb protective maternal antibodies into their circulation. In the dairy industry, calves are separated from their dams within hours after birth and undergo a weaning process starting at approximately 6 wk of age.^4^ The weaning process involves calves being transitioned from a milk-based diet onto solid feedstuffs. Calves then typically move into different environmental conditions where they interact with older animals and are exposed to new pathogens. These events in early life could influence the development of the immune repertoire.

Since the 1980s, researchers have investigated the uptake of cells from colostrum into the circulation of newborn calves.^5^ Reber et al.^6–8^ and Langel et al.^9^^,^^10^ have reported migration of colostrum-derived T cells across calves’ gut epithelium, into the circulation as potentially functional and antigen-specific T cells. Day 1 after colostrum consumption, calves fed colostrum with maternal cells had higher populations of CD4^+^CD62^+^CD45RO^-^ and CD4^+^CD62L^+^CD45RO^+^ T cells than calves fed cell free colostrum. Researchers have reported no change in the proportion of B cells in the circulation of calves after colostrum consumption.^9^ However, after vaccination, calves receiving fresh colostrum containing cells from their own dams had a higher number of B cells, compared to calves fed cell free colostrum.^10^

Although calves are born with immature immune systems, they are not entirely devoid of B cells prior to colostrum consumption.^11^^,^^12^ In the first week of life, B cells will comprise approximately 4% of the total lymphocytes in circulation, and by 6 to 8 wk of age, the percentage of B cells can reach 20%.^13^ In fetuses, IgM expressing cells have been identified as early as 59 d of gestation, and by 125 d recombination of variable (V), diversity (D), and joining (J) genes has been identified in splenic B cells.^11^^,^^14^ There is also evidence that enzymes such as activation-induced cytidine deaminase are expressed and active during this time in the spleen of the fetus.^12^ This activity would suggest the potential for somatic hypermutation (SHM) and class switch recombination (CSR) in fetal B cells prior to antigen exposure.^12^

B cells express a B cell receptor (BCR), which is a membrane-bound form of Ig, composed of 2 heavy chains (HC) and 2 light chains (LC). In the variable domains of both the HC and LC, the receptor is composed of complementarity-determining regions (CDR1, CDR2, CDR3), that are hypervariable, especially CDR3. In cattle, there are 12 functional V genes, 16 functional D genes, and 4 functional J genes.^15–18^ The recombination of VDJ gene segments determines the HC variable domain. Throughout the current study, only the CDR3 of the HC was investigated.

In humans, CDR3s are typically 5 to 20 aa in length.^19^ In contrast, in cattle the distribution is wider, including subsets of short (≤10 amino acids [aa]), medium (>10 aa and <40 aa), and ultralong (≥ 40 aa) CDR3s.^20^ Currently, the *Bos* and *Bison* genera are the only genera that have been reported to produce BCRs and antibodies with ultralong CDR3s.^21^ The ultralong CDR3 forms a unique structure with an ascending β-sheet stalk, followed by a folded knob domain and a descending stalk structure, allowing the CDR3 to reach out of the variable region structure.^22^^,^^23^ Bovine antibodies with ultralong CDR3s have been reported to have strong neutralization capabilities against experimental human viral antigens due to their structural ability to reach hidden and concave epitopes.^22–24^ There is preferential use of specific VDJ segments in the production of ultralong CDR3s, specifically *IGHV1-7*, *IGHD8-2*, and *IGHJ2-4.*^15^^,^^17^^,^^20^^,^^25^

In dairy calves, little is known about the subset of B cells expressing ultralong CDR3s and how they are influenced during immune maturation. Most studies have been completed on fetal spleen samples (∼125 d of gestation) ^11^, in the first 2 mo of life^26^, or in adult cows.^20^ Safonova et al.^27^, investigated the repertoires of 204 Black Angus beef calves (average age of 5 mo), which were raised under a different management system than dairy calves. Therefore, there is a major knowledge gap in the development and influence of age on the BCR repertoire in dairy calves in the first year of life. The variation in the percentage of ultralong CDR3 sequences, reported in the literature is wide, ranging from 0.036% to 17.02%.^15^^,^^26^^,^^28^ This variation is likely due to the effects of age, breed, and Ig isotype, as well as the diversity of laboratory methods.

The overarching objective of this study was to document changes in the IgM and IgG B cell repertoires from birth to ∼10 mo of age (∼285 d). The individual objectives include investigating how the percentage of ultralong CDR3 sequences and VDJ gene usage change within the same calf (i) from before colostrum (day 0) to after colostrum consumption (day 2), (ii) from pre-colostrum consumption (day 0) to preweaning (day 42) (iii) from preweaning (day 42) to 1 full day post-weaning (day 56), (iv) from 1 full day post-weaning (56) to after the calf has been moved into a new barn/environment (with older animals and new pathogens and microbes) (day 90), (v) from after the calf has been moved into a new environment (day 90) until the oldest sample point, to account for adjustment to housing conditions (day 285), and (vi) from before colostrum consumption (day 0) until the oldest sampling time point (day 285). These specific time points were chosen to correspond with specific ages when there is exposure to beneficial microbes as well as pathogens, in combination with stressful transitions in housing and nutrition.

It was hypothesized that the percentage of ultralong CDR3 sequences would increase after colostrum consumption, with increases in age, and with pathogen exposure. Maternal T cells and antibodies are transferred via colostrum to neonatal calves and goat kids.^9^^,^^10^^,^^29^ B cells with ultralong CDR3s have been identified as a subset of B cells in bovine colostrum.^30^ For this reason, it was hypothesized that there may be a marked increase in the percentage of ultralong CDR3s after colostrum due to the transfer of maternal components through colostrum. An increase in the percentage of ultralong CDRs was expected with an increase in age and pathogen exposure as both would allow maturation of the immune system and pathogen exposure would result in an active immune response in the calf. It was hypothesized that the percentage of ultralong CDR3s would decrease over the time of weaning. Since weaning is a stressful event for the calf, it was expected stress would lead to immunosuppression of adaptative responses. Additionally, it was hypothesized that VDJ gene usage would reflect the percentage of sequences with ultralong CDR3s.

## Materials & methods

### Animals and sampling

The Animal Care Committee of the University of Guelph approved all animal use in this study under Animal Use Protocol no. 4449. Animals were housed at the Ontario Dairy Research Centre near Elora, Ontario. Blood samples were collected on day 0, day 2, day 42, day 56, day 90, and ∼ day 285. In brief, 80 ml of whole blood was collected in K_2_EDTA coated blood tubes (Becton Dickinson, Franklin Lakes, New Jersey, USA, catalog no. 02-657-32) from Holstein dairy calves (*n* = 7) on day 0 and day 2. And 40 ml of blood was collected on day 42, day 56, day 90, and ∼ day 285. The sample collected at day 0 was collected before colostrum consumption to ensure no passive transfer had occurred prior to sampling. Calves received a 2.5% iodine navel dip immediately following calving and received an oral attenuated live bovine rotavirus-coronavirus vaccination (Calf-guard^®^, Zoetis, New Jersey, USA) following sample collection for this study but 30 min before colostrum consumption. Colostrum was not frozen or heat treated and was fed within the first 2 h of life. To be enrolled in this study the calf had to be a heifer calf that received 3 l of good quality colostrum (specific gravity >22% Brix) from its own dam from an uncomplicated birth. Following colostrum consumption calves received a vitamin E/Selenium injection. Calves were offered another 3 l of colostrum or 2 bags of colostrum replacer in 2 l of warm water (Bovine dried colostrum, Saskatoon Colostrum Company, IgG ≥ 100 g per bag) 6–12 h after the first feeding. Calves were housed in individual pens within a group nursery for the first 4 d of life and then were moved into group housing starting at day 5 of life.

The sample collected at day 2 was collected 48 h after the calf consumed at least 3 l of colostrum from its own dam. For the feedings on day 2 of life, calves were fed transition milk (second to sixth milkings) from fresh cows. Starting on day 3 and 4 of life, calves were fed milk replacer (Excel [26/18 {crude protein%/fat%}]), Grober Nutrition, Cambridge, Ontario, Canada). Calves were bottle fed 3 times a day from day 0 to day 4 and if they were strong and healthy, they were transferred to the automated feeder for 3 feedings on day 5 of life. Calves then received ad libitum access to milk replacer for the first 28 d of life, which was then reduced starting at day 28 by 0.5 l/day (12 l/day to 9 l/day). Calves also had ad libitum access to buckets of chopped straw.

Blood was collected at day 42, the day before weaning began and vaccination. On day 42 calves received an intranasal modified live virus (MLV) vaccine for bovine respiratory syncytial virus (BRSV), parainfluenza 3 virus (PI3), and bovine alphaherpesvirus 1 (infectious bovine rhinotracheitis virus [IBR]) (INFORCE3^®^, Zoetis, New Jersey, USA). The next sample was collected 1 full day after completion of the transition to solid feed (weaning), which will be referred to as day 56 (among the group the average was 59 d [55 to 60 d of age]). Calves started weaning by receiving a maximum of 9 l/d of milk replacer, which was reduced to 2 l/d by 0.5 l/d. Calves underwent a two week weaning transition, which was dependent on the day of age that the calf was deemed ready to access the automated feeder (not necessarily based on age in days). On day 63, calves received a booster dose of the intranasal BRSV/PI3/IBR MLV vaccine. In addition, on day 63, they received an intranasal avirulent live culture vaccine against *Mannheimia haemolytica* and *Pasteurella multocida* (BOVILIS^®^ ONCE PMH^®^ IN, Merck, New Jersey, USA).

At 70 to 74 d of age, heifer calves were moved into the naturally ventilated curtain-sided free stall heifer barn. The heifer barn shared space and air with older heifers and they remained in this barn until approximately 22 months of age (until ∼6 wk before calving). Blood samples were collected on day 90, after calves had been moved into the heifer barn. Between 151 and 156 d of age, calves were injected subcutaneously with a 5-way modified live viral vaccine (Pyramid5, Boehringer-Ingelheim Animal Health, Georgia, USA) for bovine viral diarrhea virus (BVDV [type 1 and 2]), IBR, PI3, BRSV. Calves would receive a booster of the Pyramid5 vaccine between 329 and 338 d of age. Three calves received a different brand of a 5-way modified live vaccine (Bovishield FP5) targeting the same 5 viruses for the booster injection. The final blood sample was collected from calves that were still on farm in March 2023 (*n* = 5) and from 1 calf in September 2022 and will be referred to as the day 285 sample. For the last blood sample calves ranged in age from 210 to 358 d of age. Only 2 of the calves in this study would have received their booster vaccination (at 330 d) at the final blood sampling timepoint. At the Ontario Dairy Research Centre, calves are continuously exposed to pathogens during development. Some of the confirmed pathogens on farm include rotavirus, coronavirus, *Cryptosporidium parvum*, *Trichophyton verrucosum* (ringworm), and pathogens involved in bovine respiratory disease. Two of the calves were treated for scours, and 3 calves were treated for pneumonia within the study period.

A plasma sample from each calf on day 0 was submitted to the Animal Health Laboratory at the Ontario Veterinary College, University of Guelph to investigate the presence of antibodies prior to colostrum consumption using a bovine respiratory serology viral neutralization panel (adenovirus 3, bovine coronavirus [(BCV], bovine viral diarrhea virus [BVDV] type 1a, BVDV type 2, IBR, BRSV, and PI3). In addition, plasma from a single calf (calf 3) was assayed by an enzyme linked immunosorbent assay (ELISA) for antibodies to bovine leukemia virus.

### Isolation of blood mononuclear cells

All centrifugation steps were performed at room temperature (RT). Whole blood was centrifuged for 15 min at 1,200×*g*, and the buffy coat was collected and suspended in 15 ml of phosphate buffered saline (PBS, Wisent Inc, Saint-Jean-Baptiste, QC, Canada, catalog no. 311-425-CL). Using an underlay of Histopaque-1077 (Sigma-Aldrich, St. Louis, Missouri, USA, catalog no. 10771) for density centrifugation, cells were centrifuged at 1,200×*g* for 15 min. The mononuclear cell portion was collected and suspended in 50 ml of PBS and centrifuged at 200×*g* for 20 min. The pellet was then washed 2 times using PBS and centrifuged at 400×*g* for 5 min. Any remaining red blood cells were lysed using sterile water; blood mononuclear cell (BMC) portions were suspended in PBS and centrifuged at 400×*g* for 5 min. The BMCs were counted, and cell viability was assessed using a Corning CytoSMART cell counter (Corning Life Sciences, Corning, New York, USA).

### Magnetic-activated positive B cell sorting (MACS)

Blood mononuclear cells (1 × 10^7^) were incubated at RT for 15 min with 250 µl of a 1/50 dilution of mouse IgG1 monoclonal anti-bovine IgM antibody (Sigma-Aldrich, catalog no. I6137, clone BM-23, 4 µg/ml) and mouse IgG1 monoclonal anti-bovine IgG antibody (Sigma-Aldrich, catalog no. B6901, clone BG-18, 5 µg/ml). Antibodies for labeling IgM^+^ and IgG^+^ B cells were included in the same reaction. Cells were washed twice with MACS buffer (PBS with 0.5% bovine serum albumin and 2 mM EDTA) and centrifuged for 5 min at 400×*g*. Next, cells were incubated in the dark at 4 °C for 15 min with rat anti-mouse IgG1 microbeads (20 μl of microbeads and 80 μl of MACS buffer for 10^7^ BMCs [Miltenyi Biotech, Bergisch Gladbach, Germany, catalog no. 130-047-102]). Cells were then washed 2 times and sorted using a positive selection method. Cells were added to a MACS mini-column (Miltenyi Biotech, catalog no. 130-042-201) per the manufacturer’s instructions. The positively (IgM^+^ and IgG^+^) sorted cells were collected and centrifuged for 5 min at 400×*g*, and the supernatant was removed. Samples were resuspended in 1 ml of TRIzol (Invitrogen, Waltham, Massachusetts, USA, catalog no. 15596026) and were stored at −80°C.

### cDNA and polymerase chain reaction (PCR)

Samples were thawed on ice, 200 μl of chloroform was added, and samples were mixed by shaking. The steps of RNA extraction are further described by Altvater-Hughes et al.^28^^,^^31^ Samples were cleaned and concentrated using the MinElute kit (Qiagen, Venlo, NL, catalog no. 74204). The quality and quantity of RNA were checked using a DeNovix Spectrophotometer/Fluorometer (DS-11) to ensure a 260 nm/280 nm absorbance ratio of ≥1.8. Using 5,000 ng of RNA and the SuperScript III First-Strand Synthesis System (Invitrogen, catalog no. 18080051), cDNA was produced. The steps of cDNA production are further described by Altvater-Hughes et al.^28^^,^^31^ Samples were stored at −20°C until they were used for downstream PCR applications.

The PCR reaction was prepared using the High-Fidelity PCR Master kit (Roche, Indianapolis, Indiana, USA, catalog no. 12140314001) and adapted from the manufacturer’s protocol. The steps of the PCR protocol are described by Altvater-Hughes et al.^28^^,^^31^ The single forward primer was used to amplify both IgM and IgG *IGHV* genes unbiasedly (Table 1). The reverse primers were used to amplify IgM or IgG heavy chains (Table 1). The PCR amplicons were cleaned and purified using the PureLink PCR Purification Kit (Invitrogen, catalog no. K310001). Quantity and quality were checked to ensure a concentration of ≥ 20 ng/μL and a 260 nm/280 nm absorbance ratio ≥1.8, and samples were stored at −20°C until sequencing was initiated.

**Table 1.** Primers and search motifs used to amplify sequences of interest and extract the CDR3.

Primer/Motif	Isotype	Nucleotide motif	Reference
Forward Primer^1^	IgM and IgG	5′-AGATGAACCCACTGTGGACC- 3′	^49^
Reverse Primer^1^	IgM	5′-TGTTTGGGGCTGAAGTCC-3′	^18^
Reverse Primer^1^	IgG	5′-GCTGTGGTGGAGGCTGAG-3′	^49^
Example Pre-CDR^2^	IgM and IgG	5′-GAGGACACGGCCACATACTACTG-3′	^28^ ^,^ ^33^
Example Post-CDR3^2^	IgM and IgG	5′-TGGGGCCAA-3′	^28^ ^,^ ^33^

1 The primers used to target the heavy chain of IgM and IgG for polymerase chain reactions.

2 Examples of the pre- and post- complementarity determining region 3 (CDR3) search motifs used to search and extract the CDR3. Motifs were designed based on notarized V and J gene segments from the ImMunoGeneTics Information System and manual curation.

### MinION sequencing

End preparation was completed by preparing 1 μg of the amplicon reaction with the NEBNext Ultra II End Repair/dA-Tailing Module (New England Biolabs, Ipswich, Massachusetts, USA, catalog no. E7546S). Samples were washed and eluted in nuclease-free water. Samples were then barcoded and prepared using the Native Barcoding Kit 24 V14 (Oxford Nanopore Technologies [ONT], Oxford, UK, catalog no. SQK-NBD114.24) and the Blunt/TA Ligase Master Mix (New England Biolabs, catalog no. M0367S). Samples were washed and eluted in nuclease-free water. Adapters were added using the NEBNext Quick Ligation Module (New England Biolabs, catalog no. E6056S) and the ONT Ligation Sequencing Kit V14 (SQK-LSK114), and then samples were washed and eluted in nuclease-free water. The barcoded samples were then added to a MinION R10.4. Flow Cell (ONT, catalog no. R10.4.1) and allowed to run for ∼72 h. Samples from each individual calf were run on separate flow cells (*n* = 7).

### Analysis

#### Cleaning

Once sequencing concluded, base calling and barcode sorting/trimming and adapter removal were completed using ONT Guppy basecaller software (v6.4.6+ae70e8f) (flowcell FLO-MIN114,—kit SQK-LSK114,—detect_barcodes,—enable_trim_barcodes) and FASTQ files containing sequence ID, phred score, nucleotide (nt) sequence, and comments were retrieved for each barcoded sample. The quality of the sequences was checked using FASTQC software (v0.11.9). The sequences were then filtered using fastp (v0.23.1) with default quality settings and –length for IgM (—length_required 700—length_limit 1200) or IgG (—length_required 200—length_limit 800). The steps describing filtering by length are further described by Altvater-Hughes et al.^28^

#### Filtering

Filtering based on sequence motifs of interest was completed using seqkit (v2.3.1) and will be referred to as the Refined Stepwise Literature-Based (RSLB) filtering method. A Nextflow (v23.04.3) pipeline was implemented to complete all steps from FASTQC to generating the final tsv file of filtered sequences without stop codons in the CDR3. All scripts for the primary analysis and RSLB filtering can be found at: https://github.com/harohodg/filtering_bovineIgM_rep and https://github.com/harohodg/filtering_bovineIgG_rep. The RSLB filtering method in this study was used to identify, extract, and provide CDR3 lengths by searching for primer sequences, IgM and IgG isotype motifs and sequences upstream and downstream of the CDR3 using regular expressions.^28^ The RSLB filtering method was developed by consulting published bovine Ig heavy chain sequences and has been refined through optimization, allowing minimal nt mismatches. An in-depth description of the filtering process for IgM and IgG sequences is described by Altvater-Hughes et al.^28^^,^^31^ A description of the primers and examples of pre- and post-CDR3 motifs can be found in Table 1. IgM and IgG amplified sequences were filtered and analyzed separately. In brief, the Nextflow pipeline identified sequences with a forward primer with up to 4 mismatches and sorted them into a file. The forward primer sorted sequences were then filtered for the inclusion of an isotype determining motif. Sequences that did not meet the filter requirements for the previous step were searched for the reverse primer with up to 4 mismatches and then converted to forward orientation. The files produced in the steps above were merged and any sequences that appeared in duplicate were removed. The generated file was then filtered using regular expressions to identify and extract the CDR3 by matching the pre-CDR3 (upstream of the CDR3) and post-CDR3 (downstream of the CDR3). After the CDR3s were identified, the length in aa was calculated from C104 (in the V region) to W118 (beginning of the J region in framework region 4), using the numbering system according to the ImMunoGeneTics Information System (IMGT).^32^ Sequences with ≥40 aa from C104 to W118 were considered to have ultralong CDR3s. Sequences with ≤10 aa were considered to have short CDR3s. The total number of sequences and sequences with ultralong CDR3s (≥40 aa) were counted. The total number of sequences with an identified CDR3 was used as the denominator to estimate the percentage of sequences with ultralong CDR3s. The extracted CDR3s were then translated and CDR3s with stop codons present were removed. Estimates that were based on sequences that had no stop codons present in the CDR3 are referred to as productive sequences in this manuscript. The percentage of ultralong CDR3s from productive sequences was calculated using the total number of productive CDR3 sequences as the denominator. It is important to highlight that all sequences with identified CDR3s in this study were analyzed, whether or not there was an ultralong CDR3 present.

### Sequence analysis

The IMGT HighV-Quest (v3.6.0) software was used to validate the filtering and ultralong CDR3 estimations.^33^ IMGT HighV-Quest provided VDJ labeling, gene usage, and CDR3 length estimation for total sequences and per clonotype.^33^ Analyzing sequences on a clonotypic basis allows compilation of the sequences that are from a single B cell clone that may have been expanded, or reads that were preferentially amplified during PCR. By definition, IMGT designated sequences as “clonotypes” if there was a unique VDJ arrangement (gene name and alleles designated by IMGT), unique in-frame CDR3 aa junction sequence, and a cysteine at location 104 and a tryptophan or phenylalanine at location 118.^34^

### Statistical analysis

An analysis of variance (ANOVA, SAS OnDemand for Academics, North Carolina, USA) was used to assess if any covariates such as the colostrum quality, colostrum volume fed, or time of feeding had an influence on the percentage of ultralong CDR3 sequences or gene usage. A non-parametric Wilcoxon signed-rank test was used to compare the differences in IgM ultralong CDR3 percentages (calculated from total sequences) and gene usage (calculated from total sequences) between pairs of critical sample points of interest (planned comparisons). The same method was applied to assess differences in IgG ultralong CDR3 percentages and gene usage between pairs of sample points of interest. The following time points were compared: before and after colostrum consumption (day 0 and day 2), before colostrum and before weaning (day 0 and day 42), before and after weaning (day 42 and day 56), after weaning and housed in a new environment (day 56 and day 90), housed in a new environment and the last sample time point (day 90 and day 285), and between birth and the last sample time point (day 0 and day 285). Significant differences were reported at *P *≤ 0.05, and tendencies were reported for *P *>* 0.05* to *<0.10*.

## Results

The covariates (colostrum quality, colostrum volume fed, time of feeding) considered in ANOVA models assessing the percentage of ultralong CDR3s or VDJ gene usage were not statistically significant. Therefore, the non-parametric Wilcoxon signed-rank test was the most suitable analysis for the planned comparisons of critical sample time points aligned with relevant biological changes related to stress, management, microbial exposure, age, and environment.

In calves, from day 0 to day 285, the number of productive IgM sequences generated from RSLB filtering, on average, was 226,455 sequences (55,563 to 489,797 sequences, data not shown). In calves, from day 0 to day 285 the number of productive IgG sequences generated from RSLB filtering for analysis, on average, was 237,420 sequences (28,669 to 572,792 sequences, data not shown). All percentages of ultralong CDR3s are based on productive sequences. Values for RSLB filtering and clonotype from DNA sequences can be found in Tables S1 and S2.

### Percentage of IgM ultralong CDR3 sequences

**Figure 1.**
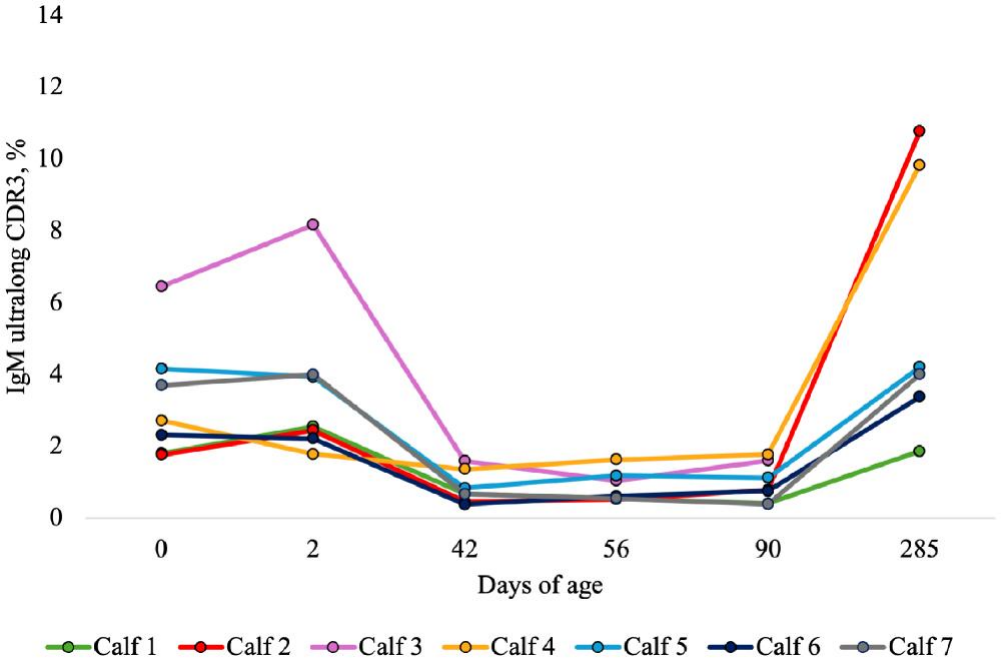
The percentages of productive IgM sequences with ultralong complementarity determining regions 3 (CDR3) from calf blood from day 0 to day 285. The percentages of productive sequences with ultralong CDR3s were generated from the RSLB filtering method and then translated from the pre-CDR3 to post-CDR3 and sequences with stop codons were removed. Samples were collected on the following days of age: 0 (before colostrum), 2 (after colostrum), 42 (before weaning), ∼56 (1 full day after weaning), ∼90 (moving into the heifer barn), and ∼285 (oldest sample point). Using a non-parametric Wilcoxon signed rank test, there was a significantly greater percentage of IgM ultralong CDR3 sequences on day 0 (3.27% ± 0.63) before colostrum consumption than day 42 (0.85% ± 0.17, *P* = 0.02) at preweaning. There was a significantly greater percentage of IgM ultralong CDR3 sequences on day 285 (5.68% ± 1.51) than day 90 (0.89% ± 0.21, *P* = 0.03). There was a significantly greater percentage of IgM ultralong CDR3 sequences on day 285 (5.68% ± 1.51) than day 0 (2.74% ± 0.41, *P* = 0.03). For statistical comparisons involving day 285, data from only the 6 surviving calves were considered.

**Figure 2.**
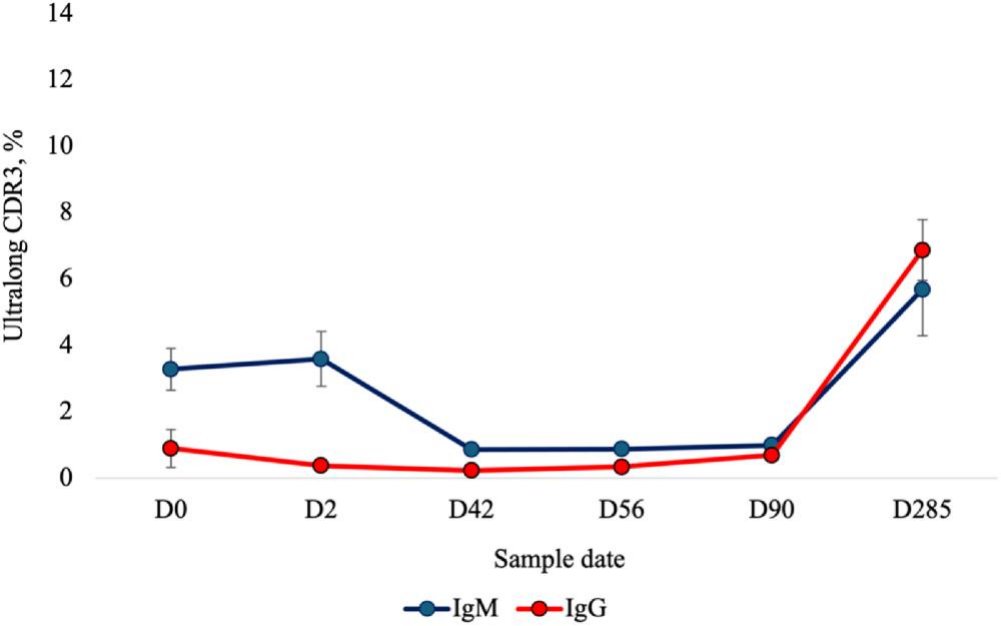
The mean percentages of productive IgM and IgG ultralong complementarity determining regions 3 (CDR3) among 7 Holstein heifer dairy calves. The percentage of IgM ultralong CDR3s were calculated from the number of the total IgM sequences. The percentage of IgG ultralong CDR3s were calculated from the number of the total IgG sequences. The estimates of the percentages of sequences with ultralong CDR3s were generated through the refined stepwise literature based (RSLB) filtering method. The percentage of productive sequences with ultralong CDR3s were generated from the RSLB filtering method and then sequences were translated from the pre-CDR3 to post-CDR3 and sequences with stop codons were removed. Samples were collected on the following days of age: 0 (before colostrum), 2 (after colostrum), 42 (before weaning), ∼56 (1 full day after weaning), ∼90 (moving into the heifer barn), and ∼285 (oldest sample point). For statistical significance of within isotype comparisons see Figs. 1 and 3.

The percentage of IgM ultralong CDR3 sequences at all time points from day 0 to day 285 are presented in Figs. 1 and 2, and Table 2. All RSLB and IMGT (total and clonotype) estimates for IgM sequences can be found in the supplementary data.

**Table 2.** The mean percentages of IgM and IgG sequences with ultralong CDR3s and gene usage of *IGHV1-7, IGHD8-2,* and *IGHJ2-4* for each sampling time point.

	Percentage use by days of age						
	Isotype (%)	0	2	42	56	90	285
Ultralong^1^ CDR3s	Mean IgM	3.27*^#^	3.58	0.85^#^	0.87	0.99^§^	5.68*^§^
	SEM	(0.63)	(0.83)	(0.17)	(0.16)	(0.20)	(1.39)
	Mean IgG	0.89*	0.37	0.23	0.34^#^	0.68^#^^§^	6.86*^§^
	SEM	(0.57)	(0.09)	(0.07)	(0.07)	(0.12)	(0.92)
*IGHV1-7* ^2^	Mean IgM	19.33*	18.96	6.18*^#^	4.91^#^^§^	4.04§^‡^	9.06^‡^
	SEM	(2.31)	(2.62)	(0.94)	(0.56)	(0.63)	(1.74)
	Mean IgG	10.85*^#^	14.46*	7.74^#^	7.03	6.78^§^	15.40^§^
	SEM	(1.10)	(2.46)	(1.17)	(0.90)	(0.51)	(2.20)
*IGHD8-2* ^2^	Mean IgM	5.19*	5.61	7.21^#^	7.82^#^^§^	8.40^§^^‡^	11.00^‡^*
	SEM	(0.98)	(0.79)	(0.26)	(0.25)	(0.28)	(0.66)
	Mean IgG	5.42*	4.34	6.48	6.66^#^	8.07^#^^§^	11.00*^§^
	SEM	(0.89)	(0.54)	(0.23)	(0.25)	(0.31)	(0.66)
*IGHJ2-4* ^2^	Mean IgM	92.52*^#^	92.57	96.44^#^^§^	96.90^§^	96.15^‡^	97.12*^‡^
	SEM	(0.66)	(0.49)	(0.23)	(0.13	(0.50)	(0.13)
	Mean IgG	89.82*	88.57	93.71	93.82^#^	94.84^#^^§^	95.42*^§^
	SEM	(1.46)	(1.44)	(0.58)	(0.42)	(0.21)	(0.26)

Statistical differences between time points for planned comparisons are highlighted using special characters. Nonparametric Wilcoxon signed rank tests were used to compare percentages.

1 The mean percentage of ultralong complementarity determining regions 3 (CDR3) from sequencing the blood IgM and IgG B cell repertoire in 7 Holstein calves. The estimates of the percentage of sequences with ultralong CDR3s were generated through the refined stepwise literature based (RSLB) filtering method. From the pre-CDR3 motif to the post-CDR3 motif, sequences were translated and sequences with stop codons were removed. Samples were collected on the following days of age: 0 (before colostrum), 2 (after colostrum), 42 (before weaning), ∼56 (1 full day after weaning), ∼90 (moving into the heifer barn), and ∼285 (oldest sample point). The mean percents for day 0 and day 90 are adjusted for comparisons with day 285 since Calf 3 was culled before day 285 due to health issues.

2 The mean percentage of *IGHV1-7*, *IGHD8-2*, and *IGHJ2-4* gene usage from sequencing the total blood IgM and IgG B cell repertoires in Holstein calves. The estimates of the gene usage were calculated using IMGT HighV Quest on a clonotypic basis. The mean percents for day 0 and day 90 are adjusted for comparisons with day 285 since Calf 3 was culled before day 285 due to health issues.

* At 2 time points indicates a significant difference between those 2 time points in the same row.

# At 2 time points indicates a significant difference between those 2 time points in the same row.

§ At 2 time points indicates a significant difference between those 2 time points in the same row.

‡ At 2 time points indicates a significant difference between those 2 time points in the same row.

From day 0 (pre-colostrum, mean: 3.27%) to day 42 (pre-weaning, 0.85%) there was a significant decrease in the percentage of productive IgM ultralong CDR3 sequences (*P* = 0.02). The percentage of productive IgM ultralong CDR3 sequences remained low, until there was a significant increase between day 90 (new environment, 0.89%) and day 285 (oldest sample point, 5.68%, *P* = 0.03). There was a significantly lower percentage of productive IgM ultralong CDR3 sequences on day 0 (2.74%, *P* = 0.03) than day 285 (5.68%). All values in this study that were compared against the day 285 sample time point were calculated for 6 calves since 1 calf was culled from the herd before the sample collection on day 285, due to chronic health issues.

### Percentage of total IgM sequences using the *IGHV1-7* gene

VDJ gene usage was analyzed among the total (quality filtered) DNA sequences for each isotype separately (IgM or IgG) and gene usage was inferred by IMGT HighV-QUEST. Gene analysis was not restricted to the ultralong CDR3 population. The *IGHV1-7* gene segment is reported to be highly used in ultralong CDR3 sequences.^15^ In the current study, the *IGHV1-7* gene segment was highly used in total IgM sequences on day 0 (mean: 19.33%, Table 2) but there was a significant decrease in the use by day 42 (6.18%, *P* = 0.02, Fig. S1). The use of *IGHV1-7* significantly decreased from day 42 (6.18%) to day 56 (4.91%, *P* = 0.02, post-weaning). The use of *IGHV1-7* continued to significantly decrease from day 56 (4.91%) to day 90 (4.21%, *P* = 0.03). The use of *IGHV1-7* then significantly increased between day 90 (4.04%) and day 285 (9.06%, *P* = 0.03). However, there was a trend for higher use of *IGHV1-7* at day 0 (19.27%) than day 285 (9.06%, *P* = 0.06).

### Percentage of total IgM sequences using the *IGHD8-2* gene

The D gene segment, *IGHD8-2*, is reported to be highly used in ultralong CDR3 sequences.^25^ On day 0, the mean percentage of the total of IgM sequences using *IGHD8-2* was 5.19%, but there were no statistically significant differences between day 0 and day 2, or between day 0 and day 42. However, there was a significant increase in the use of *IGHD8-2* from day 42 (7.21%) to day 56 (7.82%, *P* = 0.02, Table 2, Fig. S2). The percentage use of *IGHD8-2* continued to significantly increase from day 56 (7.82%) to day 90 (8.40%, *P* = 0.02). There was a significant increase in the use of *IGHD8-2* from day 90 (8.42%) to day 285 (11.00%, *P* = 0.03). The use of *IGHD8-2* on day 0 (4.30%) was significantly lower than day 285 (11.00% ± 0.66, *P*= 0.03).

### Percentage of total IgM sequences using the *IGHJ2-4* gene

The J segment, *IGHJ2-4*, is highly used in bovine VDJ sequences in general (>95%).^20^ However, in total IgM sequences, *IGHJ2-4* mean use was lower on day 0 (92.52%) and day 2 (92.57%, Table 2, Fig. S3) than in previous reports. During day 0 and day 2 samples, the gene usage was moderated by higher usage of ***IGHJ1-6*** on day 0 (5.46%) and day 2 (5.51%). There was a significant increase in the use of *IGHJ2-4* from day 0 (92.52%) to day 42 (96.44%, *P* = 0.02). There was a significant increase in the use of *IGHJ2-4* from day 42 (96.44%) to day 56 (96.90%, p = 0.03). There was a significant increase in the use of *IGHJ2-4* from day 90 (96.08%) to day 285 (97.12%, *P* = 0.03). On day 0 (92.54%), there was significantly lower usage of *IGHJ2-4* than day 285 (97.12%, *P* = 0.03). By day 285, the percentage of ***IGHJ1-6*** usage decreased to 0.91% (± 0.19).

### Percentage of the total IgG sequences with ultralong CDR3 sequences

**Figure 3.**
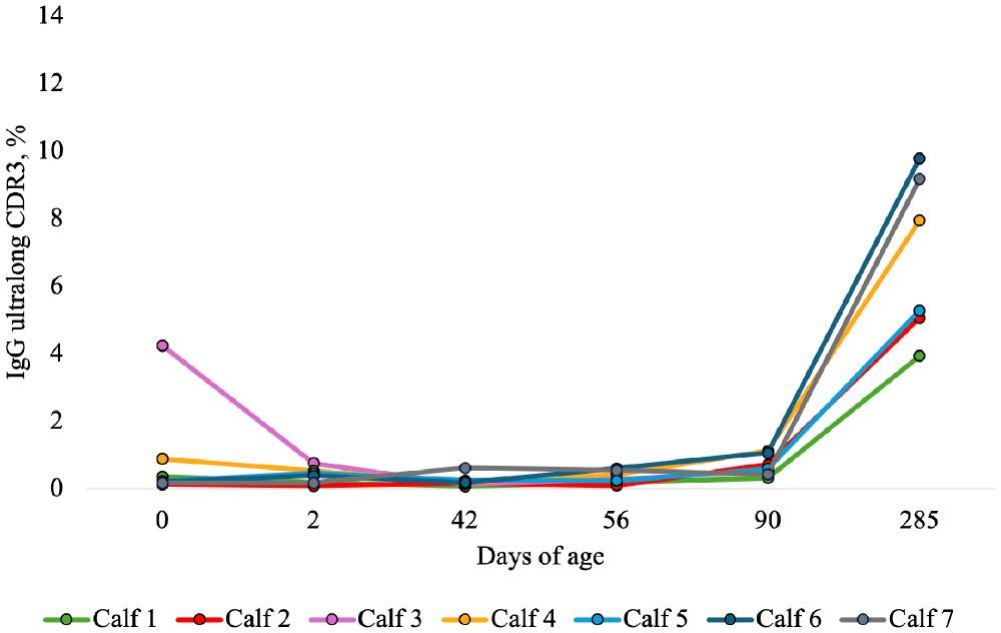
The percentages of productive IgG ultralong complementarity determining regions 3 (CDR3) calculated from the total IgG B cell repertoire. The percentages of productive sequences with ultralong CDR3s were generated from the refined stepwise literature based (RSLB) filtering method and then sequences were translated from the pre-CDR3 to post-CDR3 and sequences with stop codons were removed. Samples were collected on the following days of age: 0 (before colostrum), 2 (after colostrum), 42 (before weaning), ∼56 (1 full day after weaning), ∼90 (moving into the heifer barn), and ∼285 (oldest sample point). Using a non-parametric Wilcoxon signed rank test, there was a significantly higher percentage of IgG ultralong CDR3s on day 90 (0.68% ± 0.12) than day 56 (0.34% ± 0.07, *P* = 0.03). There was a significantly greater percentage of IgG ultralong CDR3 sequences on day 285 (6.86% ± 0.99) than day 90 (0.71% ± 0.13, *P* = 0.03). There was a significantly higher percentage of IgG ultralong CDR3 sequences on day 285 (6.86% ± 0.99) than day 0 (0.33% ± 0.12, p = 0.03). For statistical comparisons involving day 285, data from only the 6 surviving calves were considered.

The percentages of IgG ultralong CDR3 sequences at all time points from day 0 to day 285 are presented in Figs. 2 and 3, and Table 2. All RSLB and IMGT (total and clonotype) estimates for IgG sequences can be found in the supplementary data.

On day 0, prior to colostrum consumption, the mean percentage of IgG RSLB filtered productive sequences with ultralong CDR3s was 0.89% and remained <1% until after day 90. The mean percentage of productive IgG ultralong CDR3s significantly increased from day 56 (0.34%) to day 90 (0.68%, *P* = 0.03). There was a significant increase in the mean percentage of productive IgG ultralong CDR3 sequences from day 90 (0.71%) to day 285 (6.86%, *P* = 0.03). There was a significantly lower mean percentage of productive IgG ultralong CDR3 sequences on day 0 (0.33%) than day 285 (6.86%, *P* = 0.03).

On the day of birth for 1 calf (calf 3 in Table S3), 4.24% of IgG sequences had an ultralong CDR3. This calf also had the highest percentage of IgM ultralong CDR3 sequences in the first 2 d of life compared to all other calves. Calf 3 was culled from the herd due to chronic health issues (treated twice for pneumonia) at 217 d of age.

### Percentage of total IgG sequences using the *IGHV1-7* gene

VDJ gene usage was analyzed among the total (quality filtered) DNA sequences for each isotype separately (IgM or IgG) and gene usage was inferred by IMGT. Gene analysis was not restricted to the ultralong CDR3 population. The mean percentage of total IgG sequences using *IGHV1-7* was high on day 0 (Mean: 10.85%) but there was a significant increase in use by day 2 (14.46%, *P* = 0.02, Table 2, Fig. S4). There was a significant decrease in the use of *IGHV1-7* from day 0 (10.85%) to day 42 (7.74%, *P* = 0.02). There was a tendency for a decrease in the use of *IGHV1-7* from day 42 (7.74%) to day 56 (7.02%, *P* = 0.08). There was a significant increase in the use of *IGHV1-7* from day 90 (6.65%) to day 285 (15.40%, *P* = 0.03). There was a tendency for lower use of *IGHV1-7* on day 0 (10.43%) than day 285 (15.40%, *P* = 0.06).

### Percentage of total IgG sequences using the *IGHD8-2* gene

On day 0, the mean percentage of total IgG sequences using *IGHD8-2* was 5.42%, but there were no statistically significant differences between day 0 and day 2 or between day 0 and day 42 (Table 2, Fig. S5). There was a significant increase in the use of *IGHD8-2* from day 56 (6.66%) to day 90 (8.07%, *P* < 0.01). There was a significant increase in the use of *IGHD8-2* from day 90 (8.19%) to day 285 (11.60%, *P* < 0.01). There was significantly lower usage of *IGHD8-2* on day 0 (4.76%) than day 285 (11.60%, *P*= 0.03).

### Percentage of total IgG sequences using the *IGHJ2-4* gene

In IgG sequences, the use of *IGHJ2-4* was lower at day 0 (Mean: 89.82%) and day 2 (88.57%, Table 2, Fig. S6) than in previous reports. During the first 2 sample days, the gene usage was moderated by higher usage of *IGHJ1-6* on day 0 (5.58%) and day 2 (7.40%, Fig. S6). There was a tendency for an increase in the use of *IGHJ2-4* from day 0 to day 42 (93.71%, *P* = 0.08). There was a significant increase in the use of *IGHJ2-4* from day 56 (93.82%) to day 90 (94.84%, *P* = 0.02). There was a significant increase in the use of *IGHJ2-4* between day 90 (94.89%) and day 285 (95.42%, *P* = 0.03). There was significantly lower usage of *IGHJ2-4* on day 0 (89.00%) than day 285 (95.42%, *P*= 0.03). By day 285, the percentage use of *IGHJ1-6* decreased to 1.61%.

### Respiratory serology virus neutralization panel and BLV ELISA

Calf 3 was ultimately culled from the herd and had the highest percentages of IgM and IgG ultralong CDR3 sequences at birth. For this reason, samples were sent to the Animal Health Laboratory (Ontario Veterinary College, University of Guelph) to assess if there was any evidence of an antibody mediated response prior to colostrum consumption. Calf 3 had detectable viral neutralization titers to bovine coronavirus (1:16, titers <1:4 are considered negative), PI3 (1:8, titers <1:2 are considered negative), and BRSV (1:6, titers <1:2 are considered negative). The calf had no detectable viral neutralization titers to bovine adenovirus 3, BVDV type 1a, BVDV type 2, or IBR. Upon further investigation, all calves at birth (the first sample time point) had detectable plasma antibody titers to bovine coronavirus (ranging from 1:4 to 1:32). And 4 out of 7 calves had detectable titers to PI3. All calves had very low but detectable titers to BRSV; 6 out of 7 had very low but detectable titers to BVDV type 1. In brief, 3 out of 7 calves had very low but detectable titers to BVDV type 2. There were no detectable antibody titers by ELISA to bovine leukemia virus for Calf 3, and the remaining samples weren’t tested. For all titer results, see Table 3.

**Table 3.** Virus neutralization titers against common bovine viral pathogens.

	Titers						
Sample ID	BAdV-3^1^	BCV^1^	PI3^1^	BRSV^1^	BVDV type 1^1^	BVDV type 2^1^	IBR^1^
Calf 1	<1:2	1:16	<1:3	<1:4	1:3	1:3	<1:2
Calf 2	<1:2	1:32	<1:2	<1:3	1:3	1:3	<1:2
Calf 3	<1:2	1:16	1:8	1:6	<1:2	<1:2	<1:2
Calf 4	<1:2	1:8	<1:3	<1:3	1:3	<1:2	<1:2
Calf 5	<1:2	1:4	<1:3	<1:3	1:3	<1:2	<1:2
Calf 6	<1:2	1:16	<1:2	<1:3	1:2	<1:2	<1:2
Calf 7	<1:2	1:4	<1:2	<1:3	1:3	1:3	<1:2

1 BAdV-3 = Bovine adenovirus 3; BCV = Bovine coronavirus; PI3 = Parainfluenza virus 3; BRSV =  Bovine respiratory syncytial virus; BVDV type 1 & 2 = Bovine viral diarrhea; IBR = Infectious bovine rhinotracheitis (Bovine herpes virus 1).

1 For BCV, titers <1:4 are considered negative; for all other assays, titers <1:2 are considered negative.

1 Titers for all 7 calves were determined by running a virus neutralization panel run at the Animal Health Laboratory at the University of Guelph.

## Discussion

The objective of this study was to document changes in the BCR repertoire from day 0 to day 285 in Holstein heifer calves, with an emphasis on analyzing the subset of B cells expressing ultralong HC-CDR3s. It was hypothesized that the percentage of ultralong CDR3 sequences in blood would increase after colostrum consumption, because maternal lymphocytes in colostrum can cross the gut epithelium and enter a calf’s circulation. There was evidence in a previous study of higher percentages of IgG ultralong CDR3 sequences in B cells from colostrum compared to blood.^31^ The mean percentage of productive IgM ultralong CDR3 sequences on day 0 before intake of colostrum was 3.27%, which decreased by day 42 and remained low until there was an increase between day 90 and ∼day 285. The mean percentage of productive IgG ultralong CDR3 sequences was low at birth (0.89%) and remained low until there was an increase between day 90 and ∼day 285. There was no significant difference in the percentage of ultralong CDR3s between day 0 and day 2, suggesting that the number of maternal B cells in circulation may have been too low to detect, or that these cells left the circulation during this time interval and localized to secondary lymphoid organs (SLO). It is important to note that this does not mean that there was no change in the diversity of the repertoire; more extensive and intensive studies are needed for clarification.

In the fetal bovine spleen, IgM expressing cells are reportedly detected as early as 59 d of gestation,^14^ with evidence of VDJ recombination by 125 d in gestation.^11^ Early in development, the ileal Peyer’s patch is an essential site of B cell clonal expansion with evidence of mutations and ultralong CDR3s.^12^^,^^35^ Our data suggest similar events for blood-derived IgM B cells. B cells with ultralong CDR3s may move to SLO and undergo maturation, leading to the expansion in the percentage of ultralong IgM and IgG CDR3 sequences later in life. Pasman et al.^36^ reported that by 1 wk of age, the IgM repertoire is active with IgM gene transcript expression levels similar to those of adult animals. However, for IgG, it took ∼ 28 d for the repertoire gene transcript expression to become comparable to that of adult animals.^36^ Future studies should explore expression of ultralong CDR3 antibodies of the IgG1, IgG2, and IgG3 sub-isotypes as well as IgA, in blood, SLO, and mucosal sites in longitudinal studies.

On the day of birth, all calves had percentages of IgG ultralong CDR3 sequences <1%, except for 1 calf (calf 3), with 4.24% of IgG sequences having ultralong CDR3s. This calf had the highest percentage of IgM and IgG ultralong CDR3 sequences in the first 2 d of life compared to all other calves. In reviewing the health history of this animal, it was found that the calf developed pneumonia in the first week of life and again at ∼4 mo (117 d) of life and was culled from the herd by 7 mo of age. Upon further investigation, the calf was born with detectable viral neutralization titers to BCV, PI3, and BRSV; titers to PI3 and BRSV were higher compared to those of the other calves. The antibody-mediated response to these pathogens must have occurred in utero or antibodies were transferred in utero since this sample was collected prior to colostrum consumption and within the first 120 min after birth. Amoroso et al.^37^ have reported the ability of BCV to infect placental tissue in cows. Hypothetically, infection of placental tissue infection could affect the permeability of the placenta to molecules such as maternal antibodies. The finding of all calves having detectable titers of antibodies to BCV prior to colostrum consumption suggests that (i) there may have been transfer of maternal BCV specific antibodies via the placenta or, (ii) fetal calves contracted an infection with BCV in utero and mounted an immune response leading to the production of endogenous BCV specific antibodies. It is important to note that there is no placental transfer of maternal antibodies to the fetus in cattle, which does occur in humans.^2^ The discovery in newborn calves of circulating antibodies (prior to colostrum consumption) to viral pathogens, with unexpectedly high percentages of ultralong CDR3 IgG in 1 of the calves indicates a need for detailed studies of perinatal immune responses, as it challenges accepted dogmas regarding bovine neonatal antibody responses. Ultimately these findings may play an important role in how to vaccinate dams, neonatal calves, and think about maternal antibody transfer in cattle.

It was hypothesized that between day 0 and day 42, there would be an increase in the percentage of ultralong CDR3s through clonal expansion and potentially through SHM and CSR. Although the major production of ultralong CDR3s is due to specific VDJ pairing, SHM that results in a few aa additions may shift sequences classified as moderate in length to ultralong. For example, in newborn calves, CDR3 sequences of 38 aa are common and with a 2 aa addition would be subsequently classified as ultralong. Long VD junctional inserts are common in sequences from calves, that also introduce more variability in length. Additionally, CSR could result in an increase in the percentage of ultralong CDR3s since IgM ultralong CDR3s could preferentially class switch to IgG, for example. The increase from day 0 to 42 was expected due to vaccination, maturation of the immune system, and exposure to microbes and early life pathogens such as rotavirus, BCV, *Cryptosporidium parvum* and pneumonia-causing agents. However, this study showed a significant decrease in the percentage of IgM ultralong CDR3 sequences. There may be expansion events of other B cells in the calf at this point in life as the calf has experienced stressors, including dehorning, vaccination, and pressure from enteric and respiratory pathogens. There also may be a general decline in B cell response during this period for neonates, without the ability to have appropriate immune responses. Generally, there are conflicting findings on the effects of stress on immune responses. Acute stress has been reported to increase immune responsiveness, leading to increased B cell responses.^38^^,^^39^ In contrast, stress can lead to decreased immune responsiveness through decreased expression of proinflammatory components, typically associated with decreased T cell proliferation and function, which may influence the immunocompetence of the neonate.^40^^,^^41^ At the Ontario Dairy Research Centre, the weaning process has been designed to be less abrupt (the amount of milk fed to the calf is gradually decreased stepwise) than traditional weaning protocols (abrupt cessation of milk feeding to the calf). Although this weaning strategy is designed to induce less stress on the calf, we still hypothesized this change would influence immune response and result in a decrease in the percentages of ultralong CDR3s from day 42 to day 56. A study by Larsen et al.^26^ estimated that the percentage of IgG ultralong CDR3 sequences in the repertoire in calves from 1 to 2 mo of age was 0.035% (19 ultralong CDR3 sequences out of 49,945 sequences from 4 calves), similar to observed percentages for calves at this age in the current study.

From day 56 to day 90, the percentage of ultralong CDR3s was expected to increase due to increases in age, pathogen exposure, and entering a new environment, which was only seen in the percentage of IgG ultralong CDR3 sequences. The samples at this time point were collected 16 to 20 d after the calf moved into the heifer barn. Once calves are in the heifer barn of the research facilities, they are exposed to multiple pathogens, including pneumonia-causing agents and *Trichophyton verrucosum* (ringworm). Virulence factors of the fungi causing ringworm are known to have suppressive effects on immune responses.^42^ From day 90 to day 285, it was expected that with an increase in age, there would be an increase in the maturation of the immune system of the heifers and in the percentage of ultralong CDR3s. By day 285, the IgM and IgG ultralong CDR3 percentages reflected reported estimates in older animals over 1 yr of age (∼5% to 13%).^20^^,^^43^ Safonova et al.^27^ investigated the repertoire in 204 Angus cattle of approximately 5 mo of age. They reported that ultralong CDR3 percentages increased following vaccination with a 5-way multivalent modified live virus vaccine against bovine respiratory pathogens.^27^ In the current study, the increase in the percentage of ultralong CDR3s could be due to increased exposure to microbes, and/or vaccination with a 5-way modified live respiratory virus vaccine similar to that employed in the study with Angus calves,^27^ all while the heifers were settled in their new environment and within the context of increased age. Interestingly, the percentage of ultralong CDR3 and gene usage between isotype IgM and IgG followed similar patterns with an increase in the percentage of IgG ultralong CDR3s between day 90 and day 285, which may be attributed to a CSR event. More evidence through more extensive studies would be necessary to do appropriate clonotype tracking.

In contrast to the lower percentage of ultralong CDR3s in neonatal calves, the percentage usage of the *IGHV1-7* gene was high for IgM and IgG. The *IGHV1-7* gene is highly used in ultralong CDR3 sequences and typically makes up 90% to 98% of the gene usage in this subset.^44^ However, the high usage of *IGHV1-7* is not sufficient on its own to produce ultralong CDR3 sequences at birth, which is noticeable in the discrepancy between *IGHV1-7* gene usage and the percentage of ultralong CDR3 sequences. It is important to note that there is 90% sequence similarity among all bovine V gene segments, so differentiation of V segments may be difficult. The increase in *IGHV1-7* gene usage may be due to several factors including the presence of maternal cells, potential movement of certain subsets of B cells into SLO, or an expansion/reduction of specific B cell clones. Pasman et al.^36^ reported a similar expression pattern for preferential use of the *IGHV1-7* gene early in life. *IGHV1-7* is the most proximal functional V segment, which may be the most accessible segment for recombination. Preferential recombination may be due to different epigenetic factors influencing the ability to access specific gene segments.^45^^,^^46^

In this study, inference of VDJ gene usage was completed using the IMGT HighV-QUEST program. Expression of the *IGHD8-2* gene does not necessarily guarantee the production of an ultralong CDR3, but without deletions in the CDR3, *IGHD8-2* codes for 48 amino acids, leading to an ultralong CDR3. At birth, the mean percentage of IgM sequences using *IGHD8-2* was 5.19%, and for IgG, it was 5.42%. In contrast, Koti et al.^11^ report that the percentage usage of the *IGHD8-2* gene in fetal and adult spleen samples was ∼22% for both ages. In the first two months of life, the mean percentage use of IGHD8-2 in IgG sequences from blood B cells was about one-third that reported by Koti et al.^11^ for spleen tissue. There are limitations in accurately inferring D gene segment usage. Nucleotides in the CDR3 are contributed by the D gene, which is highly prone to mutations, making it difficult to infer the D gene segment of origin accurately. The *IGHD8-2* gene segment is longer than other D genes but is similar in sequence to *IGHD7-3* and *IGHD7-4*, potentially leading to D gene mislabelling. It should be noted that the *IGHD8-2* gene encodes a CPDG amino acid motif, not encoded in any other D gene segments, which provides a unique β-turn following the ascending stalk for ultralong CDR3s.^22^^,^^23^ Additionally, *IGHD1-3* is 119 nt in length, creating a CDR3 of approximately 39 amino acids and could become an ultralong CDR3 with insertions, although the structure remains undetermined to the authors’ knowledge.

In cattle, the most commonly used J gene segment is *IGHJ2-4*, which is reported to be used in >95% of total VDJ sequences.^20^ In contrast, in the current study, the gene usage profile of *IGHJ2-4* increased over the first 6 wk of life. There was a higher usage of *IGHJ1-6* in the first 2 d of life than previously reported in cattle. The *IGHJ1-6* gene segment is the most proximal J segment of the first cluster of J genes, located directly upstream of the *IGHM1* constant region.^17^ The *IGHJ2-4* is located downstream of *IGHM1* and upstream of IGHM2, with *IGHG* constant genes located further downstream. In this study by 6 wk of age, the usage of *IGHJ1-6* decreased, and the estimates of *IGHJ2-4* usage reflected what has been reported in the literature for older animals (>95%).^20^

The B cell repertoire in blood-derived B cells only represents a snapshot in time and may not be representative of tissue-resident B cell populations.^47^ At this point, it is difficult to discern whether the B cells derived from colostrum are crucial in establishing a calf’s circulating BCR repertoire. In future studies groups of negative control calves that are (i) colostrum deprived, (ii) fed colostrum replacer, (iii) fed cell-free colostrum, or (iv) fed colostrum from a different dam, would be valuable to assess whether colostral B cells truly influence the development of the repertoire. Testing calves born at the same time of the year facing the same health events would be beneficial. After maternal colostral cells are taken up from the gut into circulation, they have been hypothesized to travel to SLO to take up residence, meaning maternal B cells may be difficult to detect by 2 d of age.^48^ B cells from the dam’s colostrum may also promote B cell development in the neonate. In this study, the percentages of sequences without stop codons were analyzed rather than RSLB filtered sequences or IMGT clonotypic DNA sequences. In the future, it would be beneficial to directly sequence RNA without the use of PCR, to reduce bias in the estimates of the percentage of VDJ gene usage and ultralong CDR3 sequences. However, the same bias was inherent in all samples in this study. It would also be valuable to assess the naive antibody repertoire in the context of natural antibodies which are germline encoded with broad specificity and are present prior to antigen exposure. Changes in the naive repertoire over the first months of life should also be assessed by analyzing the expression of sub-isotypes of antibodies and their specificities.

The findings from this study provide a first glimpse of the important changes that occur in the first months of life amid important life events such as birth, colostrum consumption, weaning, vaccination, and moving to new environments with new pathogen exposure. More focused studies with more frequent collection will be critical to discern whether changes on day 2 (after colostrum intake) are due to the transfer of maternal cellular components via colostrum. It would also be beneficial to sample more frequently within the day 90 and day 285 window to better define when the percentages of ultralong CDR3s increase. Analyzing the dynamics and development of the calf B cell repertoire provides foundational knowledge on the factors that shape the repertoire of the neonatal calf until maturity. Identifying the sources of variation may clarify the influence of maternal cells on BCR repertoire development in calves, which may suggest refinements in colostrum feeding protocols, and contribute to improved vaccination strategies for neonates.

## Conclusion

There are many management and developmental changes that occur in the first few months of life for dairy calves. In this study, major shifts occur within the IgM and IgG BCR repertoires including VDJ gene usage and the expression of ultralong CDR3s in calves from birth to 10 mo of age. The expression of ultralong CDR3s decreased from 2 to 90 d of age and then increased between 90 and 285 d of age. These increases in the percentages of sequences with ultralong CDR3s may be indicative of increased antigenic exposure, increased environmental stability and concurrent maturation of immune functionality. From birth to 285 d of age, calves received colostrum, underwent weaning, received vaccinations, and commingled with older animals in new environments with new microbes and pathogens. Long-read sequencing helps to elucidate how colostrum consumption and age shape the repertoire and create diversity. At this point, little is known about the functionality and maturation of the bovine BCR repertoire after birth and the influence of maternal genetics, nutrition, and colostral components.

## Supplementary Material

vlaf079_Supplementary_Data

## Data Availability

The data underlying this article are available as Raw fastq files that can be found in the NCBI Sequence Read Archive under BioSample accession no. PRJNA1242391.
