# Supplementary material for: Expression of ultralong complementarity determining region 3 and development of IgM and IgG B cell receptor repertoires in Holstein heifer calves
Source: Immunohorizons. 2026 Jan 14;10(1):vlaf079. doi: 10.1093/immhor/vlaf079 (PMC12857222; doi:10.1093/immhor/vlaf079)
Supplement: vlaf079_Supplementary_Data [file vlaf079_supplementary_data.zip › SupplementaryInfo_Calf_10142026.pdf]

Supplementary Table 1. The mean percentage of IgM ultralong CDR3 from RSLB DNA sequences (not filtered for stop codons) and IMGT clonotype DNA sequences for each sampling time point

Day of Sample	% of Mean RSLB Filtered DNA Sequences with an Ultralong CDR3	% of Mean IMGT Filtered Clonotype DNA Sequences with an Ultralong CDR3
**0**	4.84% (± 1.00)[1]	4.00% (± 0.96)[2]
**2**	4.99% (± 1.10)	4.09% (± 0.81)
**42**	1.39% (± 0.24)	1.03% (± 0.18)
**~56**	1.43% (± 0.22)	1.02% (± 0.17)
**~90**	1.65% (± 0.28)	1.27% (± 0.22)
**~285**	8.18% (± 2.21)	6.78% (±1.69)

[1] The percentage of ultralong complementarity determining regions 3 (CDR3) from sequencing the calf blood IgM B cell repertoire. The estimates of the percentage of sequences with ultralong CDR3s were generated through the refined stepwise literature based (RSLB) filtering method and the length of the CDR3 was calculated. Samples were collected on the following days of age: 0 (before colostrum consumption), 2 (after colostrum), 42 (before weaning), ~56 (1 full day after weaning), ~90 (moving into the heifer barn), and ~285 (oldest sample point).

[2] The percentage of ultralong complementarity determining regions 3 (CDR3) from sequencing the calf blood IgM B cell repertoire using the ImMunoGeneTics (IMGT) information system. The estimates of the percentage of sequences with ultralong CDR3s were generated by calculating the percentage of sequences that were ultralong CDR3s by clonotype based on IMGT.

Supplementary Table 2. The mean percentage of IgG ultralong CDR3 from RSLB DNA sequences (not filtered for stop codons) and IMGT clonotype DNA sequences for each sampling time point

Day of Sample	% of Mean RSLB Filtered DNA Sequences with an Ultralong CDR3	% of Mean IMGT Filtered Clonotype DNA Sequences with an Ultralong CDR3
**0**	1.40% (± 0.87)[1]	2.27% (± 1.12)[2]
**2**	0.62% (± 0.12)	1.30% (± 0.15)
**42**	0.37% (± 0.07)	0.67% (± 0.09)
**~56**	0.62% (± 0.17)	0.91% (± 0.15)
**~90**	1.01% (± 0.17)	1.52% (± 0.27)
**~285**	9.55% (± 1.22)	11.48% (±1.80)

[1] The percentage of ultralong complementarity determining regions 3 (CDR3) from sequencing the calf blood IgM B cell repertoire. The estimates of the percentage of sequences with ultralong CDR3s were generated through the refined stepwise literature based (RSLB) filtering method and the length of the CDR3 was calculated. Samples were collected on the following days of age: 0 (before colostrum consumption), 2 (after colostrum), 42 (before weaning), ~56 (1 full day after weaning), ~90 (moving into the heifer barn), and ~285 (oldest sample point).

[2] The percentage of ultralong complementarity determining regions 3 (CDR3) from sequencing the calf blood IgM B cell repertoire using the ImMunoGeneTics (IMGT) information system. The estimates of the percentage of sequences with ultralong CDR3s were generated by calculating the percentage of sequences that were ultralong CDR3s based on a clonotypic level.

Supplementary Table 3a. The individual percentages of productive IgM and IgG sequences with ultralong CDR3s

			**Sample time point (days)**					
	**Isotype**	**Calf**	**0**	**2**	**42**	**~56**	**~90**	**~285**
**Ultralong CDR3s**[1]	**IgM**	1	1.80[1]	2.55	0.67	0.54	0.50	1.86
		2	1.76	2.44	0.46	0.52	0.78	10.77
		3	6.45	8.17	1.59	1.04	1.60	N/A
		4	2.72	1.79	1.36	1.63	1.77	9.83
		5	4.17	3.93	0.84	1.18	1.12	4.21
		6	2.32	2.22	0.38	0.61	0.75	3.38
		7	3.70	4.00	0.67	0.55	0.39	4.01
		**Mean**	3.27	3.58	0.85	0.87	0.99	5.68
		**Std**	1.67	2.19	0.46	0.43	0.53	3.69
	**IgG**	1	0.36[1]	0.19	0.07	0.18	0.32	3.93
		2	0.13	0.09	0.16	0.09	0.72	5.06
		3	4.24	0.75	0.13	0.27	0.51	N/A
		4	0.88	0.53	0.19	0.42	1.12	7.95
		5	0.22	0.46	0.24	0.23	0.60	5.27
		6	0.20	0.39	0.18	0.60	1.07	9.79
		7	0.17	0.18	0.62	0.54	0.43	9.17
		**Mean**	0.89	0.37	0.23	0.34	0.68	6.86
		**Std**	1.50	0.23	0.18	0.19	0.31	2.43

[1]The individual percentages of ultralong complementarity determining regions 3 (CDR3) from sequencing the blood IgM and IgG B cell repertoire in 7 Holstein calves. The estimates of the percentage of sequences with ultralong CDR3s were generated through the refined stepwise literature based (RSLB) filtering method. From the pre-CDR3 motif to the post-CDR3 motif, sequences were translated and sequences with stop codons were removed. Samples were collected on the following days of age: 0 (before colostrum), 2 (after colostrum), 42 (before weaning), ~56 (1 full day after weaning), ~90 (moving into the heifer barn), and ~285 (oldest sample point).

Supplementary Table 3b. The individual percentages of IGHV1-7, IGHD8-2, and IGHJ2-4 in IgM and IgG sequences for each sampling time point

	Isotype	Calf	Sample time point (days) 0	2	42	~56	~90	~285
IGHV1-7[1]	IgM	1	16.64	29.72	5.22	4.72	3.24	4.44
		2	13.59	13.95	2.73	2.39	2.24	13.86
		3	19.70	26.85	10.15	6.55	5.74	NA
		4	15.98	13.09	7.30	6.4	6.54	14.72
		5	15.71	13.12	4.09	3.83	3.23	6.41
		6	22.01	18.93	5.87	4.83	3.82	6.37
		7	31.67	30.19	7.93	5.65	5.16	8.54
		Mean	19.33	20.84	6.18	4.91	4.28	9.06
		Std	6.11	7.89	2.50	1.47	1.56	4.26
	IgG	1	7.26	9.39	6.66	6.31	5.15	7.11
		2	11.21	12.33	3.94	4.24	6.38	13.89
		3	13.40	17.76	8.89	7.99	7.56	NA
		4	8.46	10.52	7.53	6.68	8.45	18.01
		5	7.86	8.23	4.22	4.32	5.05	12.23
		6	13.18	15.97	11.29	8.94	8.06	21.93
		7	14.59	26.99	11.66	10.69	6.79	19.23
		Mean	10.85	14.46	7.74	7.02	6.78	15.40
		Std	2.99	6.52	3.09	2.37	1.34	5.39
IGHD8-2[1]	IgM	1	3.37	4.93	6.78	7.31	7.99	9.27
		2	4.25	4.85	7.12	7.72	8.58	13.03
		3	10.57	10.13	7.38	7.81	8.25	NA
		4	6.54	5.87	8.59	9.12	9.74	12.97
		5	4.43	4.70	7.26	8.01	8.73	10.04
		6	3.62	3.80	6.41	6.97	7.30	9.91
		7	3.56	4.96	6.96	7.79	8.20	10.77
		Mean	5.19	5.61	7.21	7.82	8.40	11.00
		Std	2.60	2.08	0.69	0.67	0.75	1.62
	IgG	1	6.88	4.33	5.95	5.84	7.36	9.05
		2	5.00	4.37	6.19	6.07	7.72	10.73
		3	9.33	4.27	5.97	6.03	7.33	NA
		4	5.66	6.93	6.68	7.43	9.65	12.74
		5	5.69	4.93	6.20	6.93	7.67	10.95
		6	3.14	3.22	7.68	7.34	8.46	13.24
		7	2.21	2.34	6.70	6.96	8.29	12.90
		Mean	5.42	4.34	6.48	6.66	8.07	11.60
		Std	2.35	1.43	0.61	0.66	0.82	1.63
IGHJ2-4[1]	IgM	1	93.99	92.50	96.58	96.53	96.15	96.86
		2	94.33	93.45	97.49	97.59	97.24	97.47
		3	92.43	90.68	95.46	96.66	96.58	NA
		4	91.23	92.75	96.29	96.62	95.40	96.60

		5	93.84	94.68	96.46	96.98	96.64	97.49
		6	89.48	91.48	96.07	96.97	97.49	97.09
		7	92.36	92.49	96.709	96.95	93.58	97.19
		**Mean**	92.52	92.58	96.44	96.90	96.15	97.12
		**Std**	1.74	1.29	0.62	0.36	1.33	0.35
	**IgG**	1	92.61	83.47	94.49	93.98	94.77	96.30
		2	92.08	91.78	93.64	93.19	95.20	95.48
		3	94.73	84.42	91.46	91.79	94.54	NA
		4	88.23	91.90	94.69	94.24	95.03	95.26
		5	91.02	92.42	95.32	94.97	95.36	95.41
		6	83.84	86.13	91.71	93.54	93.77	94.25
		7	86.22	89.89	94.64	95.01	95.18	95.83
		**Mean**	89.82	88.57	93.71	93.82	94.84	95.42
		**Std**	3.87	3.81	1.53	1.12	0.55	0.68

[1]The individual percentages of *IGHV1-7*, *IGHD8-2*, and *IGHJ2-4* gene usage from sequencing the blood IgM and IgG B cell repertoires in Holstein calves. The estimates of the gene usage were calculated using IMGT on a clonotypic basis. Samples were collected on the following days of age: 0 (before colostrum), 2 (after colostrum), 42 (before weaning), ~56 (1 full day after weaning), ~90 (moving into the heifer barn), and ~285 (oldest sample point).

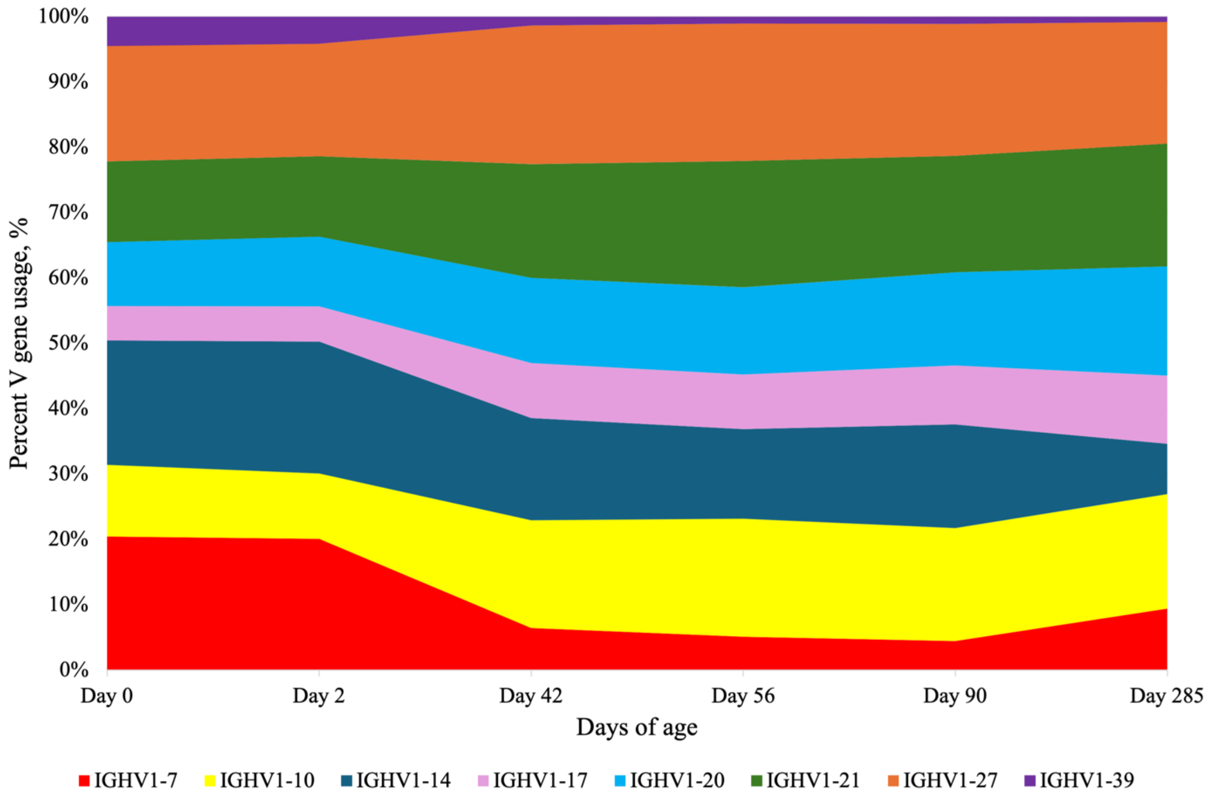

Supplementary Figure 1. The mean percent of IgM *IGHV* gene usage from day 0 to ~ day 285 from 7 Holstein heifer calves, only 6 calves contributed to the estimates of gene usage on day 285. The most highly used 8 V genes are displayed as the mean V gene usage for the 7 calves at each timepoint. The ImMunoGeneTics (IMGT) platform was used to label V genes and usage was calculated on a clonotype basis. In red is the use of *IGHV1-7*, which is the gene preferentially used for ultralong complementarity determining region 3 sequences. There was significantly greater usage of *IGHV1-7* on day 0 (19.33% ± 2.31) than day 42 (6.18% ± 0.94, p=0.02). There was significantly greater usage of *IGHV1-7* on day 42 (6.18% ± 0.94, preweaning) than day 56 (4.91% ± 0.56, p=0.02, post-weaning). There was significantly higher usage of *IGHV1-7* in IgM sequences at day 56 (4.91% ± 0.56) than day 90 (4.04% ± 0.63, p=0.03). There was significantly higher usage of *IGHV1-7* in IgM sequences at day 285 (9.06% ± 1.74) than day 90 (4.04% ± 0.63, p=0.03). There was a trend for decreased percent usage of *IGHV1-7* at day 285 (9.06% ±1.74) than day 0 (19.27% ± 2.73, p=0.06).

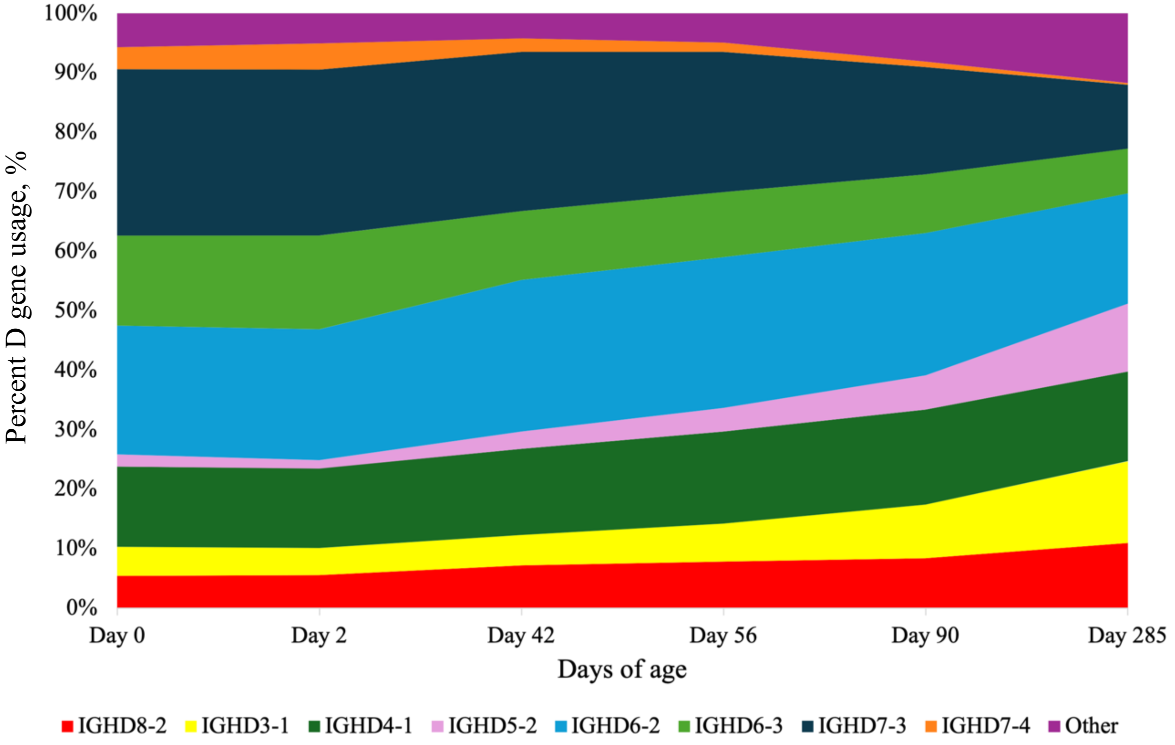

Supplementary Figure 2. The mean percent of IgM *IGHD* gene usage from day 0 to ~ day 285 from 7 Holstein heifer calves, only 6 calves contributed to the estimates of gene usage on day 285. The most highly used 8 D genes are displayed as the mean D gene usage for the 7 calves at each timepoint. The ImMunoGeneTics (IMGT) platform was used to label D genes and the percentage was calculated on a clonotype basis. In red is the use of *IGHD8-2*, which is the gene preferentially used for ultralong complementarity determining region 3 sequences. There was significantly higher use of *IGHD8-2* on day 56 (7.82% ± 0.25) than day 42 (7.21% ± 0.26, p= 0.02). There was significantly higher use of *IGHD8-2* on day 90 (8.40% ± 0.28) than day 56 (7.82% ± 0.25, p= 0.02). There was significantly higher use of *IGHD8-2* on day 285 (11.00% ± 0.66) than day 90 (8.42% ± 0.33, p=0.03). There was significantly higher use of *IGHD8-2* on day 285 (11.00% ± 0.66) and day 0 (4.30% ± 0.48, p=0.03).

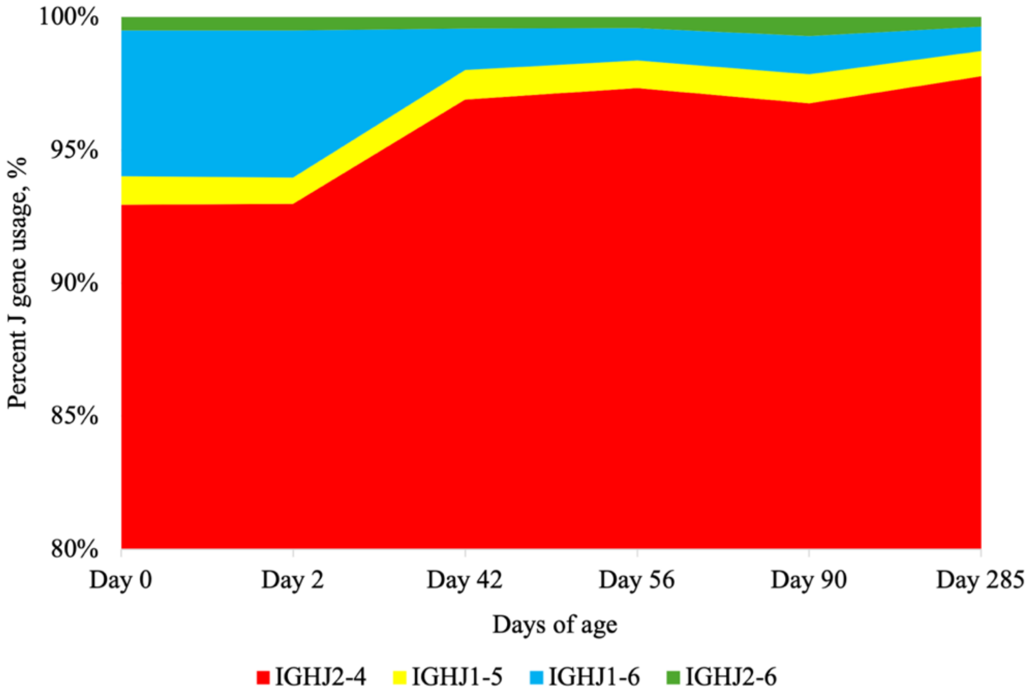

Supplementary Figure 3. The mean percent of IgM *IGHJ* gene usage from day 0 to ~ day 285 from 7 Holstein heifer calves, only 6 calves contributed to the estimates of gene usage on day 285. The most highly used 4 J genes are displayed as the mean J gene usage for the 7 calves at each timepoint. The ImMunoGeneTics (IMGT) platform was used to label J genes and percentage was calculated on a clonotype basis. In red is the use of *IGHJ2-4*, which is the gene preferentially used for all sequences and sequences with ultralong complementarity determining region 3. There was a significant increase in *IGHJ2-4* from day 0 to day 42 and day 285. The y-axis begins at 80% since the major gene of use (*IGHJ2-4*) is typically used in more than 90% of sequences. By day 42, there was a significantly higher percent usage of *IGHJ2-4* (96.44% ± 0.23) than day 0 (92.52% ± 0.66, p=0.02). There was significantly higher usage of *IGHJ2-4* on day 56 (96.90% ± 0.13, post-weaning) than day 42 (96.44% ± 0.23, preweaning, p=0.03). There was significantly higher usage of *IGHJ2-4* by day 285 (97.12% ± 0.14) than day 90 (96.08% ± 0.59). On day 285 (97.12% ± 0.14), there was significantly higher usage of *IGHJ2-4* than day 0 (92.54% ± 0.78, p=0.03).

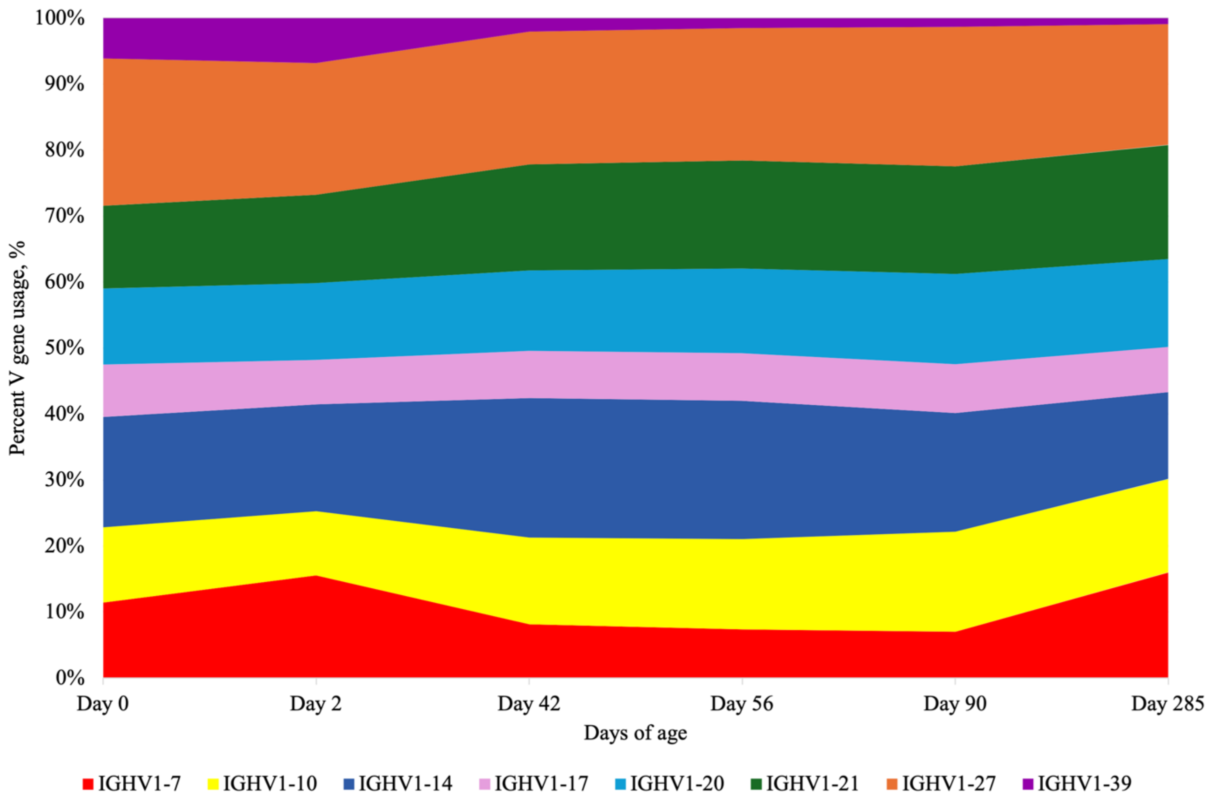

Supplementary Figure 4. The mean percent of IgG *IGHV* gene usage from day 0 to ~ day 285 from 7 Holstein heifer calves, only 6 calves contributed to the estimates of gene usage on day 285. The most highly used 8 V genes are displayed as the mean V gene usage for the 7 calves at each timepoint. The ImMunoGeneTics (IMGT) platform was used to label V genes and usage was calculated on a clonotype basis. In red is the use of *IGHV1-7*, which is the gene preferentially used for ultralong complementarity determining region 3 sequences. The usage of *IGHV1-7* was high on day 0 (10.85% ± 1.13) but there was a significant increase in usage by day 2 (14.46% ± 2.46, p=0.02). There was significantly greater *IGHV1-7* usage on day 0 (10.85% ± 1.13) than day 42 (7.74% ± 1.16, p=0.02). There was a tendency for higher usage of *IGHV1-7* on day 42 (7.74% ± 1.17) than day 56 (7.03% ± 0.90, p=0.08). There was significantly higher usage of *IGHV1-7* on day 285 (15.40% ± 2.20) than day 90 (6.65% ± 0.58, p=0.0313). There was a tendency for higher usage of *IGHV1-7* on day 285 (15.40% ± 2.20) than day 0 (10.43% ± 1.24, p=0.06).

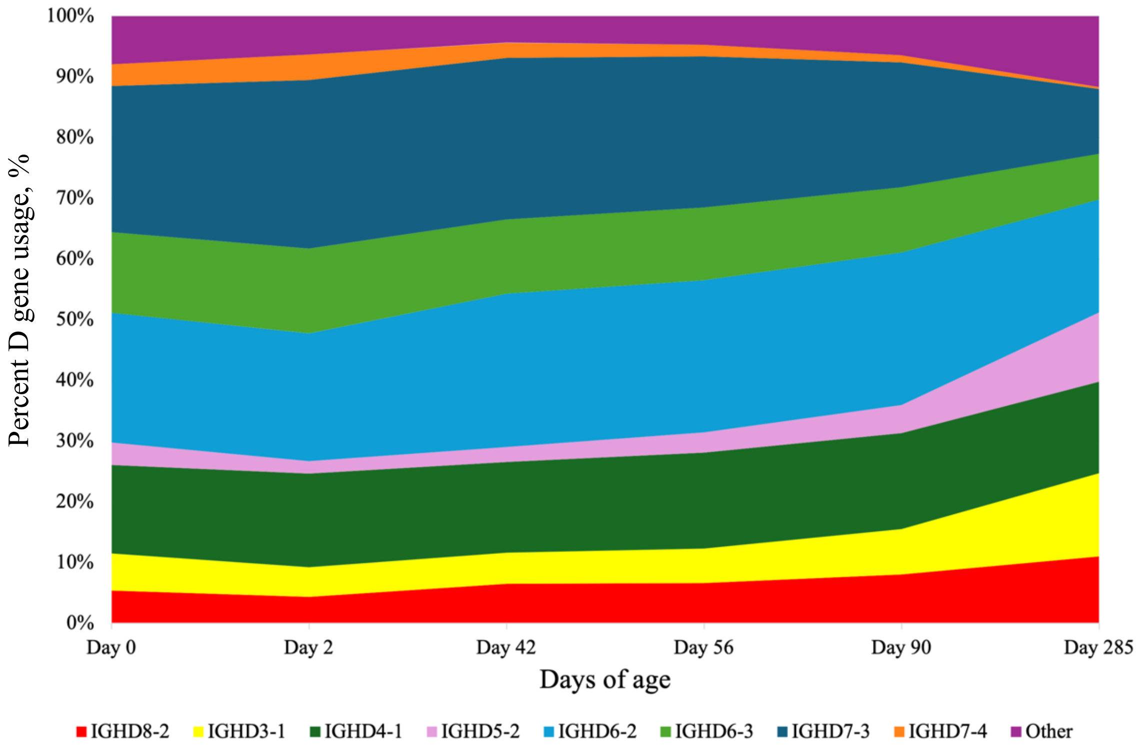

Supplementary Figure 5. The mean percent of IgG *IGHD* gene usage from day 0 to ~ day 285 from 7 Holstein heifer calves, only 6 calves contributed to the estimates of gene usage on day 285. The most highly used 8 D genes are displayed as the mean D gene usage for the 7 calves at each timepoint. The ImMunoGeneTics (IMGT) platform was used to label D genes and percentage was calculated on a clonotype basis. In red is the use of *IGHD8-2*, which is the gene preferentially used for ultralong complementarity determining region 3 sequences. There was significantly higher use of *IGHD8-2* on day 90 (8.07% ± 0.31) than day 56 (6.66% ± 0.25, $p<0.01$). There was significantly higher use of *IGHD8-2* on day 285 (11.60% ± 0.67) than day 90 (8.19% ± 0.34, $p<0.01$). There was significantly higher use of *IGHD8-2* on day 285 (11.60% ± 0.67) than day 0 (4.76% ± 0.72, $p= 0.03$).

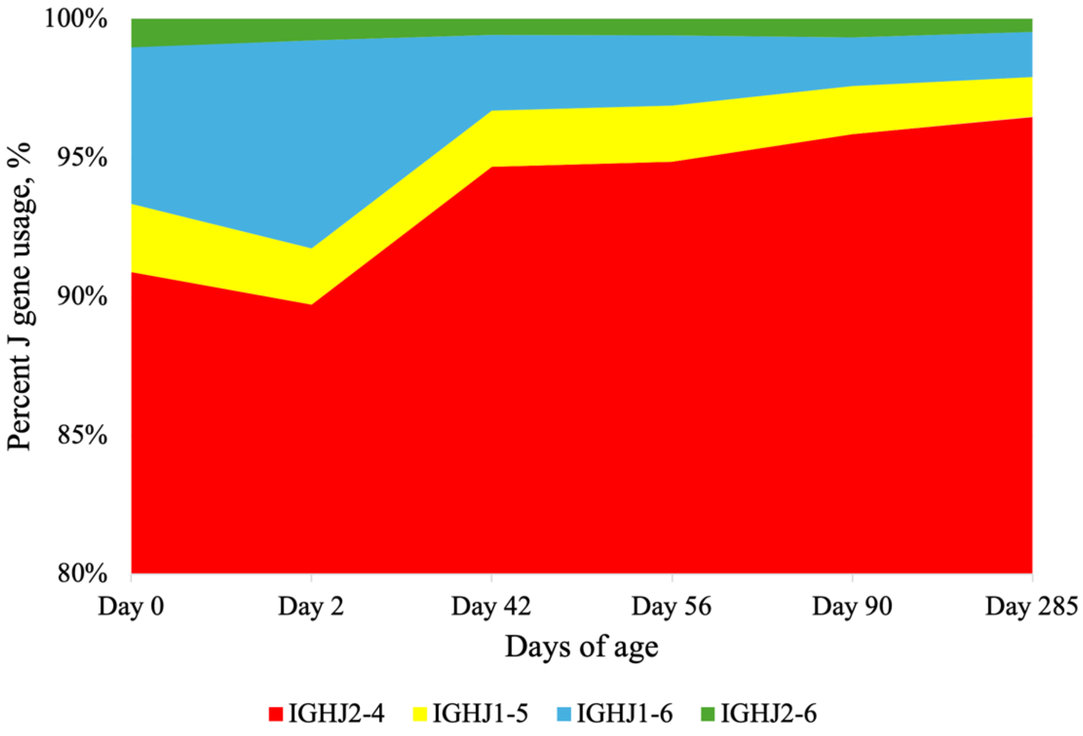

Supplementary Figure 6. The mean percent of IgG *IGHJ* gene usage from day 0 to ~ day 285 from 7 Holstein heifer calves, only 6 calves contributed to the estimates of gene usage on day 285. The most highly used 4 J genes are displayed as the mean J gene usage for the 7 calves at each timepoint. The ImMunoGeneTics (IMGT) platform was used to label J genes and percentage was calculated on a clonotype basis. In red is the use of *IGHJ2-4*, which is the gene preferentially used for all sequences and sequences with ultralong complementarity determining region 3. The y-axis begins at 80% since the major gene of use (*IGHJ2-4*) is typically used in more than 90% of sequences. There was a tendency for a higher percent usage of *IGHJ2-4* on day 42 (93.71% ± 0.58) than day 0 (89.82% ± 1.46, p=0.08). There was significantly higher usage of *IGHJ2-4* on day 90 (94.84% ± 0.21) than day 56 (93.82% ± 0.42, p=0.02). There was significantly higher usage of *IGHJ2-4* on day 285 (95.42% ± 0.28) than day 90 (94.89% ± 0.24). There was significantly higher usage of *IGHJ2-4* on day 285 (95.42% ± 0.28) than day 0 (89.00% ± 1.43, p=0.03).
